# Correlates of women’s intentions to be screened for human papillomavirus for cervical cancer screening with an extended interval

**DOI:** 10.1186/s12889-016-2865-8

**Published:** 2016-03-02

**Authors:** Gina S. Ogilvie, Laurie W. Smith, Dirk van Niekerk, Fareeza Khurshed, Heather N. Pedersen, Darlene Taylor, Katharine Thomson, Sandra B. Greene, Suzanne M. Babich, Eduardo L. Franco, Andrew J. Coldman

**Affiliations:** University of British Columbia, Vancouver, BC Canada; Women’s Health Research Institute, Vancouver, BC Canada; British Columbia Cancer Agency, Vancouver, BC Canada; British Columbia Centre for Disease Control, Vancouver, BC Canada; Gillings School of Global Public Health, University of North Carolina Chapel Hill, Chapel Hill, NC USA; Richard M. Fairbanks School of Public Health, Indiana University-Purdue University Indianapolis, Indianapolis, IN USA; McGill University, Montreal, QC Canada; BC Women’s Hospital and Health Centre, Room H203G, 4500 Oak Street, Vancouver, BC V6H 3 N1 Canada

**Keywords:** Cervical cancer, Human papillomavirus, Theory of planned behaviour, Screening programs

## Abstract

**Background:**

High-risk HPV DNA testing has been proposed as a primary tool for cervical cancer screening (HPV-CCS) as an alternative to the Papanicolaou cytology- method. This study describes factors associated with women’s intentions to attend cervical cancer screening if high-risk HPV DNA testing (HPV-CCS) was implemented as a primary screening tool, and if screening were conducted every 4 years starting after age 25.

**Methods:**

This online survey was designed using the Theory of Planned Behaviour to assess factors that impact women’s intentions to attend HPV-CCS among women aged 25–69 upon exit of the HPV FOCAL trial. Univariate and regression analyses were performed to compare the demographic, sexual history, and smoking characteristics between women willing and unwilling to screen, and scales for intention to attend HPV-CCS. A qualitative analysis was performed by compiling and coding the comments section of the survey.

**Results:**

Of the 981 women who completed the survey in full, only 51.4 % responded that they intended to attend HPV-CCS with a delayed start age and extended screening interval. Women who intended to screen were more likely to have higher education (AOR 0.59, 95 % CI [0.37, 0.93]), while both positive attitudes (AOR 1.26, 95 % CI [1.23, 1.30]) and perceived behavior control (AOR 1.06, 95 % CI [1.02, 1.10]) were significant predictors of intention to screen. Among women who provided comments in the survey, a large number of women expressed fears about not being checked more than every 4 years, but 12 % stated that these fears may be alleviated by having more information.

**Conclusions:**

Acceptability of increased screening intervals and starting age could be improved through enhanced education of benefits. Program planners should consider measures to assess and improve women’s knowledge, attitudes and beliefs prior to the implementation of new screening programs to avoid unintended consequences.

## Background

Globally, cervical cancer is the fourth most common type of cancer in women [1]. In high income countries, implementation of cytology programs for cervical cancer screening (CCS) have led to significant reductions in cervical cancer incidence and mortality [2]. However, there are limitations in the screening performance of cytology including its low sensitivity which means testing must be repeated every two or 3 years, and high cost of infrastructure for implementing and maintaining programming quality [3–5]. It is now well established that high-risk types of human papillomavirus (HR-HPV) are the cause of cervical cancer [6–8], and the use of HR-HPV DNA testing as a primary tool for cervical cancer screening (HPV-CCS) has been recommended [9–14]. As a more sensitive yet less specific test, it is proposed that HR-HPV DNA testing be used as the first-line test, with those HPV positive receiving second line triage tests with higher specificity, such as cytology or HPV genotyping [11–13, 15–17].

Clinical trials have confirmed increased detection of pre-cancerous lesions with the use of HR-HPV DNA testing compared to cytology [18–20]. A recent pooled-analysis by Ronco et al. found that HPV-CCS provided 60–70 % greater protection against invasive cervical cancers compared to cytology [15]. Their findings also demonstrated that the efficacy of HPV-CCS is maximized by extending the screening interval to every 5 years for HPV negative women, and with screening beginning after the age of 30 years [15, 21]. Similarly, an American study of over 42,000 women concluded that HPV primary testing with reflex genotyping in women starting at age 25 years was as effective as a hybrid screening strategy of cytology or co-testing while using fewer screening tests [17]. While the use of HPV as the primary tool for CCS may offer improved detection of precancerous lesions and invasive cancer, the switch from cytology to HPV may have unintended consequences on women’s participation in cervical cancer screening programs due to changes as a result of the use of HPV testing [22]. HPV is a sexually acquired virus, and its use as a test may change a ‘blameless’ screen for cancer into one where patients start to focus on the sexual transmissibility of HPV [23–25]. It is also uncertain whether women will accept changes to screening regimens, including delayed starting age and extended intervals.

Currently in British Columbia (BC), Canada, screening begins at age 21, or approximately 3 years after sexual debut, and screening is recommended every 2 years. In our previous study, women’s intention to be screened with HPV decreased from 84.0 to 51.4 % when they were advised about the delayed start for screening and the extended interval [22]. In this follow-up analysis we will explore the demographic and risk factors, as well as underlying constructs of behavior associated with women’s intention to be screened for cervical cancer when the age to start screening is delayed until age 25 and the screening interval is extended to 4 years.

## Methods

### Objectives

The goal of this study was to determine the factors associated with women’s intentions to be screened for cervical cancer if HR-HPV DNA testing was used instead of Pap smears when screening was conducted every 4 years and screening started at 25 years of age.

### Participants

Survey participants were recruited at study exit from the HPV FOCAL trial (ISRCTN79347302). HPV FOCAL is a clinical trial determining the efficacy of HPV based primary screening to liquid based cytology (LBC) for the detection of cervical intraepithelial neoplasia grade 2 or greater (CIN2+). Trial methods are described in detail elsewhere [26, 27] but briefly, women in BC aged 25–65 eligible for routine cervical cancer screening were recruited through the provincial Cervical Cancer Screening Program by collaborating family physicians and randomized to receive either HR-HPV DNA testing or LBC. Women were provided study information prior to enrollment, along with the consent form [26]. The information package provided to participants included detailed description of Pap screening in BC, HPV and its association with cervical cancer,, HR-HPV testing, the study purpose and the differences between the study arms and the follow-up algorithms in each of these study arms. Participants and their family physicians were both initially blinded to study arm allocation since randomization occurred when samples were received at the laboratory. Although study allocation was never expressly stated to participants, there were opportunities throughout the study where women could have become aware of their study allocation based on duration of follow up, or information provided by their family physician.

Participants were invited to complete the survey at exit if they had completed the study per protocol [26]. At the time the data was abstracted, primarily participants in the safety arm were eligible to complete the survey. Women in the safety arm received HR-HPV DNA testing at baseline, and exited the study 2 years later with LBC testing 2 years later. Additionally women from the control arm, the safety arm, and the intervention arm who had been discharged from the study early due to colposcopy findings were also invited to complete the survey.

### Survey design

At study exit, women were invited via email to complete an electronic survey. Women were provided with background information on HPV, transmission, and a rationale for the use of primary HR-HPV DNA testing including an extended screening interval (Appendix 1). The survey was based on *Theory of Planned Behaviour* (TPB) [28], which has been widely used to understand factors associated with conducting a specific health behaviour [29]. It examines the most proximate determinant of the health behaviour, the intention to conduct the behaviour as well as the key constructs underlying the behaviour. As part of the TPB, three particular constructs are examined: knowledge and attitudes to the health behaviour, ability to control access to the behaviour (perceived behavioural control), and key personal and institutional influencers in the decision to engage in the health behaviour (direct and indirect subjective norms). In addition, we examined the role of partners regarding HPV test results. Items related to these constructs were examined individually, and then combined into a scale. Survey questions corresponding to each scale are detailed in Table 1. Item correlation in the scale is determined using Cronbach’s alpha, and if *p* < 0.5 then items within the scale are dropped until acceptable agreement is achieved. The details of how scale consistency was achieved for the questions used in this survey, and scale characteristics are detailed elsewhere [22]. Participants were provided a space at the end of the survey to include any comments. Ethics approval for this study was obtained from the University of British Columbia Research Ethics Board (H06-04032).

**Table 1:** Characteristics of scale items

Screening concepts	Scale items
Attitudes to HPV testing every 4 years and after age of 25	A22. Having an HPV test to screen for cervical cancer every 4 years and after age of 25 instead of a Pap smear every year would be: •Accurate •Safe •Protect my health •Acceptable
Subjective Norms: Direct	SND 2: Most people who are important to me would think that I should have an HPV test to screen for cervical cancer instead of a Pap smear SND3: People who are important to me would expect me to have an HPV test to screen for cervical cancer instead of a Pap smear SND4. I would feel under social pressure to have an HPV test to screen for cervical cancer instead of a Pap smear SND4: I would feel under social pressure to have an HPV test to screen for cervical cancer instead of a Pap smear
Subjective Norms: Indirect	SNI5. My family physician would think that I should have an HPV test to screen for cervical cancer instead of a Pap smear SNI6. What my family physician thinks is important to me SNI7. My friends would think that I should have an HPV test to screen for cervical cancer instead of a Pap smear SNI8. What my friends think is important to me SNI9. My spouse/partner would think that I should have an HPV test to screen for cervical cancer instead of a Pap smear SNI10. What my spouse/partner thinks is important to me SNI11. The BC Cancer Agency would recommend that I should have an HPV test to screen for cervical cancer instead of a Pap smear SNI12. What the BC Cancer Agency recommends is important to me
Role of partners	CP13. If I had a cervical cancer screening result that showed I had an HPV infection, I would feel comfortable sharing the results with my partner(s) CP14. My spouse would be understanding if I had an HPV infection
Perceived behavioral control [22]	PBC15. I am confident that I could have an HPV test to screen for cervical cancer instead of a Pap smear PBC16. For me to have an HPV test to screen for cervical cancer instead of a Pap smear would be PBC17. Whether or not I would have an HPV test to screen for cervical cancer instead of a Pap smear would be entirely up to me PBC18. How much control would you have over whether you had an HPV test to screen for cervical cancer instead of a Pap smear?

### Analysis

Univariate and regression analyses were performed using SAS (v. 9.3.1) to compare the characteristics of survey responders to non-responders, in addition to presenting demographic, sexual history, smoking data, and scales for intention to attend HPV-CCS. All variables that reached significance of *p* ≤ 0.2 were considered for stepwise logistic regression modeling [30]. A logistic regression model was created to determine predictors of women’s intentions to participate in a screening program grounded in a new paradigm with HPV, extended intervals and delayed start, with odds ratios (OR) and 95 % confidence intervals (CI) to measure the strength and precision of associations. Responses from the comments section were compiled and coded and grouped into themes in NVivo (v 10) by a research assistant who was not previously involved in survey development, survey administration, data collection, data abstraction or analysis. Themes were independently reviewed and revised by a senior research staff. Themes that related to the extended screening interval or delayed age to start screening were summarized for this study.

## Results

In total, 2016 email invites were sent to women who had exited the HPV FOCAL trial. 1538 received the survey, 1466 unique women clicked onto the survey, and 981 completed the entire survey (Table 2). There were no demographic or risk behavior differences between survey respondents and non-respondents [22]. A total of 51.4 % (504/981) women responded that they intend to participate in HPV-CCS with a delayed start of age 25 and an extended screening interval (Table 3).

**Table 2:** Summary of survey responses collected

Category	Count	Percentage (%)
Number of invites	2016	
Number of clicks on survey (received)	1538	100
Number of unique respondents	1446	94.02
Number of respondents who did not submit survey as complete	191	12.42
Number of respondents with duplicated complete surveys	3	0.19
Number of respondents who did not answer all questions	250	16.25
Number of respondents with eligible results	1094	71.13
Number of unique respondents with eligible results	1094	71.13
Number of eligible respondents who answered all short EPI-Q	981	63.78

**Table 3:** Univariate analysis of demographic factors, risk factors, and scales for intention to attend for HPV-CCS – “I would be willing to have an HPV test to screen for cervical cancer after the age of 25 and every 4 years instead of a Pap smear every year after becoming sexually active”

Variable	Group	Overall	Agree	Disagree	*p*-value^a^
Overall	N(%)	981(100 %)	504(51.4 %)	477(48.6 %)	
Marital Status	Divorced	108(11.0 %)	54(10.7 %)	54(11.3 %)	0.46
	Common law/married	689(70.2 %)	363(72.0 %)	326(68.3 %)	
	Never married	112(11.4 %)	53(10.5 %)	59(12.4 %)	
	Widowed	7(0.7 %)	5(1.0 %)	2(0.4 %)	
	Missing	65(6.6 %)	29(5.8 %)	36(7.5 %)	
Education	High school or less	133(13.6 %)	54(10.7 %)	79(16.6 %)	0.007
	More than high school	848(86.4 %)	450(89.3 %)	398(83.4 %)	
Sexual Partners - Ever	0	1(0.1 %)	1(0.2 %)	0	0.42
	1	185(18.9 %)	96(19.0 %)	89(18.7 %)	
	2–5	364(37.1 %)	193(38.3 %)	171(35.8 %)	
	6–10	221(22.5 %)	110(21.8 %)	111(23.3 %)	
	11–50	198(20.2 %)	101(20.0 %)	97(20.3 %)	
	>50	12(1.2 %)	3(0.6 %)	9(1.9 %)	
Ethnic Origin	Chinese	81(8.3 %)	37(7.3 %)	44(9.2 %)	0.47
	Aboriginal	24(2.4 %)	11(2.2 %)	13(2.7 %)	
	Other	876(89.3 %)	456(90.5 %)	420(88.1 %)	
Smoke, Ever	No	627(63.9 %)	331(65.7 %)	296(62.1 %)	0.24
	Yes	354(36.1 %)	173(34.3 %)	181(37.9 %)	
Age, Recruitment	Median(IQR)	45(38–53)	45(38–54)	44(38–51)	0.07
Attitudes to HPV testing (A1)	Mean score (SD)	19.5(7.1)	23.6(5.1)	15.2(6.3)	<0.001
Subjective Norms, Direct (SND2-3)	Mean score (SD)	11.0(2.6)	11.5(2.5)	10.5(2.6)	<0.001
Subjective Norms, Indirect (SNI5-12)	Mean score (SD)	34.8(31.9)	42.2(30.2)	27.0(31.9)	<0.001
Perceived Behavioural Control (PBC15-18)	Mean score (SD)	23.4(4.1)	24.2(3.8)	22.6(4.3)	<0.001
Contacting Partners (CP13-14)	Mean score (SD)	12.6(2.2)	12.7(2.2)	12.5(2.3)	0.33

^a^ based on either chi-square or Kruskal-Wallis test as appropriate on valid data only

The results of the univariate analysis are captured in Table 3. Women with more than high school education were more likely to intend to attend screening with HPV with extended interval and delayed start dateagreed they would attend screening (89.3 % vs 83.4 %, *p* = 0.007). No other demographic characteristics (ie. age at recruitment, marital status, number of lifetime sexual partners, ethnicity, or ever smoked cigarettes) were statistically significant. All scale items for attitudes, subjective norms (direct & indirect), and perceived behavior control were significantly associated with intention to screen with HPV with an extended interval and delayed start date (*p* < 0.001), while items assessing the impact of partners were not significant (*p* = 0.33).

In the final logistic model, women with the intention to screen at age 25 and every 4 years were more likely to be educated beyond high school (AOR 0.59, 95 % CI [0.37, 0.93]) (Table 4). Both positive attitudes regarding the value of HPV testing (AOR 1.26, 95 % CI [1.23, 1.30]), and perceived behavior control (AOR 1.06, 95 % CI [1.02, 1.10]) were also significant predictors of intention to screen in this scenario (Table 4). Direct and indirect subjective norms were not significant predictors of intention to screen in this model.

**Table 4:** Adjusted odds ratio estimates^a^ for factors associated with intention to attend for HPV-CCS

Dependent variable	Independent variables	Adjusted OR	95 % CI
“I would be willing to have an HPV test to screen for cervical cancer after the age of 25 and every 4 years instead of a Pap smear every year after becoming sexually active”	Attitudes to HPV testing every 4 years after the age of 25 (A22)	1.26	(1.23, 1.30)
	Perceived Behavioural Control (PBC15-18)	1.06	(1.02, 1.10)
	Education	0.59	(0.37, 0.93)

^a^ Stepwise logistic regression was used to select final variables from full multivariable model (variables with *p* value ≤0.2 in univariate analysis were considered for multivariable model)

Among 981 women who completed the survey, 316 (32 %) participants provided comments: 14 from the control arm, 284 from the safety arm and 18 from the intervention arm. The mean age for these women is 46. They were predominantly married (67 %), half of whom were university graduates or had a university advanced degree (52.2 %). Twenty-two percent of all comments were related to the extended screening interval. While some women stated that the 4-year interval would be an improvement over screening every year, a large number of women expressed fears about not being checked more often than every 4 years. This was especially true of older women who have historically been told to have a Pap yearly for most of their lives. As one participant stated, “I worry that only being tested every 4 years gives plenty of time for issues to arise and go untreated”. Seven percent of women who commented expressed concerns over the delayed screening age, for example one participant stated “Age (25) is too late. I had a 19 year old staff member with cervical cancer. Girls have sex as early as 14…11 years is too long to wait”. Another stated “How did you come up with the every 4 years and starting at 25 years old? Should it not be more frequent at a younger age? It just seems to be an arbitrary number”. Twelve percent of the comments suggested that their fears about the new HPV testing technology and the extended interval may be alleviated by having more information.

## Discussion

Many countries are considering, or have confirmed implementation of HPV testing as the primary method for cervical cancer screening including the United States (U.S.), Australia, and Canada [31–36]. The higher sensitivity and negative predictive value compared to cytology leads to higher detection of pre-cancerous lesions, and lower incidence of cervical cancer which is why the screening interval can be safely extended [15]. In this study, participants of HPV FOCAL [22, 26, 27] completed a survey based on the Theory of Planned Behaviour to assess women’s intention to attend HPV-CCS, which uses scales for attitudes, direct and indirect subjective norms, and perceived behaviour control. The goal was to assess factors related to intention to attend HPV testing as the primary method for CCS if screening were conducted every 4 years and starting at age 25. Although most women expressed intention to attend HPV-CCS, willingness decreased substantially when coupled with the extended screening interval and delayed screening start (84.2 to 54.2 %, to 51.4 % respectively). Unfavorable beliefs about extending the screening interval were also observed in studies in the U.S. among women of menopausal age enrolled in an HPV testing clinical trial [37], and among low income women [38]. In our study, most demographic factors were not significant predictors of willingness to attend screening, while positive predictors were attitudes regarding the value of HPV testing, perceived behavior control, and higher education.

The lower acceptability of the extended screening interval could lead to unintended consequences and impacts on screening programs. Women may choose to return for screening every 2 to 3 years instead of the recommended 4 to 5 years. This could lead to over-screening and potentially over-treatment for cervical lesions that could resolve without treatment. In studies conducted in the U.S., the majority of women surveyed preferred to screen annually despite knowledge of changes to guidelines recommending an extended screening interval [37, 38]. In both studies the physician’s recommendation was an important influence on acceptability and patient behavior [37, 38]. These and other studies have shown that patient expectations or anxieties influence the screening recommendations of health care providers, and that this often results in over-screening in women [37–41]. Some reasons women have continued annual visits include a desire for routine checkup, other gynecologic or health concerns, to maintain relationships with their physician, and reassurance of continued health [37]. Other studies in the U.S. have noted that physicians often provide screening more frequently than new guidelines recommend, either on patient request, or out of concern that patients would not attend annual health exams unless cervical cancer screening was included [37, 39, 42]. Over-screening would increase probability of a false positive result, which leads to iatrogenic harm such as anxiety, and over-treatment. It would also use valuable health care resources unnecessarily. Similarly, if women attend screening before the recommended age of 25 or 30 years, or more frequently than recommended, the chances are higher HPV test with higher sensitivity would detect transient infections that would otherwise regress on their own. Programs must anticipate women’s potential responses and concerns with significant changes such as these, and should ensure robust planning and education to mitigate any negative impact on screening attendance or health service resource utilization.

It has been reported elsewhere that the adverse emotional responses experienced by women are directed at the HPV infection itself rather than HPV testing [43]. Feelings of stigma, embarrassment and anxiety of having a sexually transmitted infection could be exacerbated in the context of an older age to start screening or delayed screening interval. Efforts to increase patient’s knowledge of cervical cancer screening and HPV infection to dispel myths and misconceptions, normalize HPV, and improve attitudes are widely recommended by researchers [37, 38, 43]. Our study population is a cohort of educated women who participated in a clinical trial, the vast majority of whom received both cytology and HR-HPV DNA testing for cervical cancer screening, at different points during trial participation. Overall, the results of this survey suggest that attitudes and beliefs held by women would likely influence their willingness to participate in HPV-CCS in the event that the screening interval was extended and age of starting screening were increased. These findings have important programmatic implications with regard to health information communication and knowledge translation. Health care providers will play a pivotal role in communicating to their patients changes in cervical cancer screening programs. Providers need to be supportive of changes to programs, and relay information appropriately to ensure patients are at ease with a transition to HPV-CCS. Public health leaders should carefully consider how messages are communicated to the public and to practitioners as changes are implemented to ensure acceptance and uptake.

### Limitations

Not all email addresses to which the survey was sent were functional, and the overall response rate was 63 %, therefore, selection bias is possible. However, our previous study [22] demonstrates that respondents and non-respondents did not differ significantly in demographic or risk factors, which may indicate that our sample was representative of the FOCAL study population, which in itself was representative of the provincial population targeted for cervical cancer screening. The vast majority of women were from the safety arm, and therefore would have received both cytology and HPV testing at different times in the study, however a very small proportion of the population who were discharged early did not receive HPV-CCS. Although detailed study information including HPV-CCS was provided at the time of enrollment and consent, it is difficult to determine how well participants understood this information, or how much they recalled after 2 years when the survey was administered. It is also possible that despite having participated in HPV-CCS for the study, women still may not have known specifically what type of testing their samples received in the study, and may have incorrectly assumed they were HPV negative or positive, depending on how their provider conveyed results to them. Knowledge of their results may have had an impact on how they answered questions in the survey. Women participating in FOCAL were recruited from two main geographic areas in BC, and therefore, this study may not be generalizable to all women in BC. It should also be noted that the women who provided comments may over represent women who were concerned about screening and may not be representative of all women who were eligible to participate in the survey.

## Conclusion

As programs begin planning for implementation of HR-HPV DNA testing as the primary tool for cervical cancer screening, it becomes important to assess the acceptability and willingness of this screening technology among women currently engaged in screening programs. This study assessed women’s intention to attend HPV-CCS, using scales for attitudes, direct and indirect subjective norms, and perceived behaviour control based on the Theory of Planned Behaviour. All women were provided information about HPV and HPV testing at when they consented to participate in HPV FOCAL. Our results demonstrated that women’s intention to attend HPV-CCS decreased significantly with the introduction of the longer screening interval and the delayed starting age for screening. Attitudes toward HPV-CCS, and perceived behavior control were particularly important factors in women’s intention to attend screening. This has important implications for program planners who are considering changes to screening programs and want to avoid unintended consequences such as reducing the uptake of screening, or over-screening. Our findings also indicate a need for robust and targeted messaging and education on HPV and HPV based CCS for women and healthcare providers.

### Availability of data and materials

Data requests can be directed to the corresponding author.

## Appendix

### Background information provided prior to study questionnaire

Thanks for participating in the HPV FOCAL trial. We invite you to complete this on-line survey in order to help us to plan for the future of cervical cancer screening in British Columbia. We are conducting this survey to help understand women’s attitudes to screening for cervical cancer with HPV testing instead of pap smears. This survey will take you about 10 min to complete, and all who complete the survey are eligible to win one of 5 iPODs. Please remember, your name, or any other personal identifiers are not linked with the questionnaire responses in any way.

Here is some background information for you to consider before you complete this survey.

The human papillomavirus (HPV) is a common virus that can infect the cervix (part of a woman’s womb). It is now known to be the cause of cervical cancer. Women develop HPV infections in the cervix after having sexual activity with a partner who is infected with HPV. However, HPV is so common that over 75 % of sexually-active women will have an HPV infection of their cervix sometime during their life. Most women who find out they have an HPV infection in the cervix after the age of 30, were infected with HPV years before. Over 90 % of women who are infected with HPV in the cervix get rid of the infection naturally. It is only women who have longstanding infections with certain types of HPV who may be at risk for developing cervical cancer. Women may not have known it in the past, but it is these same HPV infections that are the most common reason for abnormal Pap smears.

Right now in BC, women start cervical cancer screening once they become sexually active. We now know that testing for HPV infections in the cervix is more accurate than the Pap smear for predicting whether or not a woman will develop cervical cancer, So, in BC, women would be screened every 4 years with HPV testing instead of every year with Pap screening.
